# Stable, Monodisperse, and Highly Cell-Permeating Nanocochleates from Natural Soy Lecithin Liposomes

**DOI:** 10.3390/pharmaceutics11010034

**Published:** 2019-01-16

**Authors:** Martina Asprea, Francesca Tatini, Vieri Piazzini, Francesca Rossi, Maria Camilla Bergonzi, Anna Rita Bilia

**Affiliations:** 1 Department of Chemistry, University of Florence, Via U. Schiff 6, 50019 Sesto Fiorentino, Florence, Italy; aspreamartina@gmail.com (M.A.); vieri.piazzini@unifi.it (V.P.); mc.bergonzi@unifi.it (M.C.B.); 2 Institute of Applied Physics “N. Carrara” (IFAC-CNR), Via Madonna del Piano 10, 50019 Sesto Fiorentino, Italy; f.tatini@ifac.cnr.it (F.T.); f.rossi@ifac.cnr.it (F.R.)

**Keywords:** soy lecithin liposomes, nanocochleates, andrographolide, freeze-drying, gastrointestinal stability, uptake and safety

## Abstract

(1) Background: Andrographolide (AN), the main diterpenoid constituent of *Andrographis paniculata*, has a wide spectrum of biological activities. The aim of this study was the development of nanocochleates (NCs) loaded with AN and based on phosphatidylserine (PS) or phosphatidylcholine (PC), cholesterol and calcium ions in order to overcome AN low water solubility, its instability under alkaline conditions and its rapid metabolism in the intestine. (2) Methods: The AN-loaded NCs (AN–NCs) were physically and chemically characterised. The in vitro gastrointestinal stability and biocompatibility of AN–NCs in J77A.1 macrophage and 3T3 fibroblasts cell lines were also investigated. Finally, the uptake of nanocarriers in macrophage cells was studied. (3) Results: AN–NCs obtained from PC nanoliposomes were suitable nanocarriers in terms of size and homogeneity. They had an extraordinary stability after lyophilisation without the use of lyoprotectants and after storage at room temperature. The encapsulation efficiency was 71%, while approximately 95% of AN was released in PBS after 24 h, with kinetics according to the Hixson–Crowell model. The in vitro gastrointestinal stability and safety of NCs, both in macrophages and 3T3 fibroblasts, were also assessed. Additionally, NCs had extraordinary uptake properties in macrophages. (4) Conclusions: NCs developed in this study could be suitable for both AN oral and parental administration, amplifying its therapeutic value.

## 1. Introduction

The design and production of appropriate drug delivery systems, in particular, nanosized ones, offer an advanced approach to optimised bioavailability and/or the stability of drugs, to control drug delivery and to maintain drug stability during transport to the site of action. A successful drug carrier system should possess a long shelf life, optimal drug loading and release properties, and exert a much higher therapeutic efficacy as well as have low side effects [1,2].

Phospholipids are the main amphiphilic components of the cell membrane and currently represent the main constituents of nanovectors because they can self-assembly in aqueous milieu, generating different supramolecular structures such as micelles and vesicles [1,3]. Typically, their variation in head groups, aliphatic chains and alcohols leads to a wide variety of phospholipids, generally classified as glycerophospholipids and sphingomyelins. The most common natural glycerophospholipids are phosphatidylcholine (PC), phosphatidylinositol, phosphatidylserine (PS), phosphatidylglycerol and phosphatidic acid, having diverse acyl moieties, principally myristoyl, palmitoyl, oleoyl and stearoyl.

**Figure 1:**
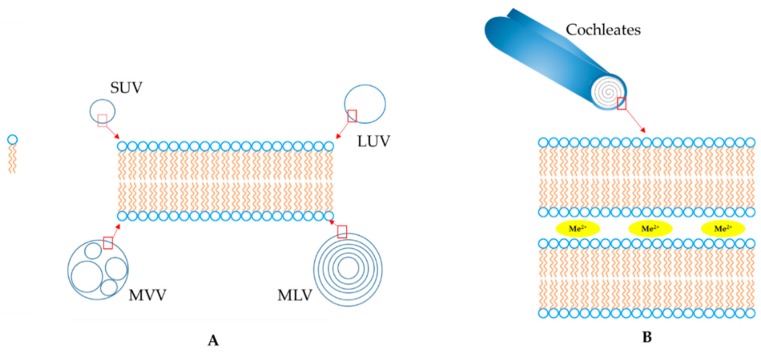
Schematic representation of the structures of liposomes (**A**) and nanocochleates (**B**).

In particular, glycerophospholipids are the specific constituents of liposomes, which are widely used as drug vectors because of their high biocompatibility, non-toxicity, complete biodegradability, and non-immunogenic effects after both systemic and non-systemic routes of administration [4]. Conversely, the therapeutic use of vesicles has some limitations, principally poor stability and availability under the harsh conditions typically presented in the gastrointestinal tract [1,2,5,6]. A very limited number of studies report on the use of cochleates as an alternative platform to vesicles in order to overcome these limitations. Cochleates were first observed by Verkleij et al. [7] using phosphatidylglycerol liposomes and later by Papahadjopoulos et al. [8], using phosphatidylserine liposomes in the presence of divalent metal cations (Me^2+^), i.e., Ca^2+^, Ba^2+^, Fe^2+^, Mg^2+^ and Zn^2+^. Cochleates can be produced as nano- and microstructures and they are extremely biocompatible, with excellent stability due to their unique compact structure. They present an elongated shape and a carpet roll-like morphology always accompanied by narrowly packed bilayers, through the interaction with Me^2+^ as bridging agents between the bilayers (Figure 1). During this arrangement, the close approach of bilayers is dependent on dehydration of the head group of the phospholipid. They roll-up in order to minimise their interaction with water and, consequently, cochleates possess little or no aqueous phase. The relevant differences between cochleates and different liposomes, i.e., small unilamellar vesicle (SUV), large unilamellar vesicle (LUV), multilamellar vesicle (MLV) and multivesicle vesicle (MVV), are reported in Figure 1.

The bilayers in a cochleate are organised very precisely at a very close repeating distance of 54 Angstrom [9] with a water-free interior, which is a rigid, stable, rod-shaped structure. Due to this unique structure, cochleates can be easily lyophilised to a free-flowing powder that can be incorporated in capsules for oral administration or re-dispersed in water for parental administration. Yet what remains very unclear is their mechanism of permeation throughout the biological membranes. It is reported that after oral administration, cochleates cross the epithelium, delivering the loaded drug into the blood vessel [10]. There are two current hypotheses to explain the mechanism of permeation. According to the first assumption, the contact of the calcium-rich membrane of the cochleate with a cell can cause a perturbation and the reordering of the cell membrane. Subsequently, there is fusion between the outer layer of the cochleate and the cell membrane [10]. An alternative hypothesis for the delivery mechanism of cochleates is phagocytosis. In both cases, once within the interior of a cell, a low calcium concentration results in the opening of the cochleate crystal and the release of the entrapped drug [11,12,13].

Currently, cochleates represent difficult drug delivery systems for clinical use, principally due to the numerous difficulties in producing monodisperse systems because of a tendency to form stable and huge aggregates, which represent a serious drawback at the industrial level. Diverse patents and publications have reported different strategies to overcome these limitations [11], in particular, the use of methylcellulose, casein, or albumin, but proteins may decrease stability and safety due to the change of pharmacokinetic parameters. Methylcellulose is able only in part to disrupt the formed aggregates. Other natural polysaccharides (including celluloses, gums, and starches) have been recommended as inhibitors of the aggregation processes, but their efficiency still remains ambiguous [11,12]. In recent times, the ability of citric acid to remove Ca^2+^ ions from the external surface of cochleates, leading to the dispersion of the aggregates, has been investigated [13]. Furthermore, a recent approach compared a novel microfluidics-based strategy with the conventional cochleate production methods; however, the formation of aggregates was still present in the samples [14].

The aim of this study was the production of monodisperse and stable nanocochleates (NCs) using two different phospholipids, PC and PS, loaded with a typical small hydrophobic natural product, andrographolide (AN) from the Asiatic medicinal plant *Andrographis paniculata*. Besides the numerous potential activities ranging from anti-inflammatory to neuroprotection, antidiabetic to anti-obesity properties, and antitumor activity to hepatoprotective activity [15], AN has poor water solubility (3.29 ± 0.73 µg at 25 °C) [16], which deeply limits its biodistribution and localisation, resulting in low bioavailability [17]. Additionally, AN is unstable in gastrointestinal media and has a very short biological half-life (*t*½ = 1.33 h) after a single oral dose [18]. The stability of developed nanocochleates after lyophilisation and in simulated gastrointestinal fluids was investigated. In addition, the possible hazards and the cellular effects of NCs were determined using J774a.1 murine macrophages and 3T3 fibroblasts. Lastly, studies on uptake using a confocal microscope were carried out in the macrophages cell line.

## 2. Materials and Methods

### 2.1. Materials

The phospholipon 90G (soy phosphatidylcholine, PC) was sourced from the Italian agent AVG srl (Milan, Italy) of Lipoid AG (Cologne, Germany). The dioleoyl phosphatidylserine (PS) was a kind gift from Lipoid AG (Cologne, Germany). The following reagents were from Sigma-Aldrich (Milan, Italy): pepsin from porcine gastric mucosa, bile salts, andrographolide (AN), fluorescein isothiocyanate (FITC, purity ≥ 90%, HPLC), lipase from porcin pancreas, sodium hydroxide (NaOH), calcium chloride (CaCl_2_), cholesterol, phosphate buffered saline (PBS) bioperformance certified, paraformaldehyde (PFA), Dulbecco’s Modified Eagle Medium (DMEM), fetal bovine serum (FBS), l-glutamine, penicillin–streptomycin solution, WST-8 kit, acetonitrile (HPLC grade), methanol (HPLC grade), formic acid (analytical grade), hydrochloric acid (HCl) (analytical grade) and dichloromethane (CH_2_Cl_2_). The water used was from the Milli-Q_plus_ system from Millipore (Milford, CT, USA). The phosphotungstic acid (PTA) was from Electron Microscopy Sciences (Hatfield, PA, USA). The dialysis kit was from Spectrum Laboratories, Inc. (Breda, The Netherlands). The J774a.1 murine macrophages and the 3T3 fibroblasts were purchased from the American Type Culture Collection (ATCC^®^ TIB-67™, Manassas, VA, USA). A LT-4000 reader from Labtech was used to read the absorbance (Bergamo, Italy).

### 2.2. Preparation of PC- and PS-based Liposomes and NCs

The NCs were obtained from nano-sized liposomes (LPs), which were prepared according to the film hydration method [19]. The liposomes were formulated as follows: the required amounts of phospholipids (60 mg) and cholesterol (20 mg) were dissolved in a dichloromethane/methanol mixture (20 mL of a mixture, 3:2 *v*/*v*). The obtained organic solution was evaporated under vacuum and the lipid film was hydrated by the addition of PBS (10 mL) using a mechanical stirrer (RW20 digital, IKA, Staufen im Breisgau, Germany) for 30 min in a water bath at a constant temperature of 37 °C for PC and 60 °C for PS. The resulting formulations were optimised by ultrasonication (3 min, two cycles of 90 s) in an ice bath to prevent lipid degradation. Subsequently, a gentle centrifugation (1205× *g*, 1 min) was performed to remove possible metallic particles released during the ultrasonication. The NCs were prepared from the nanoliposomes according to the trapping method, described by Asprea et al. [20]. Briefly, a 0.1 M solution of CaCl_2_ was added drop-by-drop to the liposomal suspension under magnetic stirring (150 rpm, room temperature) until the formulation appeared cloudy, indicating the formation of NCs. The molar ratio between PC and CaCl_2_ was 1:1, while the molar ratio between PS and CaCl_2_ was 1:4.

### 2.3. Characterisation of Nanocarriers: Size, Polydispersity Index and ζ-Potential

The Zsizer Nano series ZS90 (Malvern Instruments, Malvern, UK) outfitted with a JDS Uniphase 22 mW He-Ne laser operating at 632.8 nm, an optical fiber-based detector, a digital LV/LSE-5003 correlator and a temperature controller (Julabo water-bath) set at 25 °C was used for Dynamic Light Scattering (DLS) measurements, including for the particle size, polydispersity index (PdI) and ζ-potential. The cumulant method was used to analyse time correlation functions, obtaining the mean diameter of the nanocarriers (Z-average) and the size distribution using the ALV-60X0 software V.3.X provided by Malvern. The size characterisation technique for the nanoparticles in suspension, based on the measurement of their translational diffusion coefficient, related to the length, L, of their major axis is as

(1) $$D=\;\frac{kBT}{3\pi \eta L}\;FD,$$

where *η* represents the viscosity of the solvent, *kB* represents the Boltzmann constant and *T* represents the sample temperature. *FD* is a geometrical coefficient depending on the shape, but not the size, of the particles [21,22]. In particular, for NCs, the expressions of *FD* corresponding to these particle shapes are

*FD* = log*ρ* + 0.312 + 0.565/*ρ* − 0.1/*ρ*^2^, (2)

*ζ*-potential values were obtained from the electrophoretic mobility, using the Henry correction to Smoluchowski’s equation. The samples were diluted in distilled water and an average of three measurements at the stationary level were taken. A Haake temperature controller kept the temperature constant at 25 °C.

### 2.4. Morphological and Size Characterisation by Transmission Electron Microscopy (TEM)

A transmission electron microscope (TEM, Jeol Jem 1010, Tokyo, Japan) was used to evaluate the morphology, shape and dimensions of NCs. The NCs dispersion was diluted 10-fold and placed on a carbon film-covered copper grid and stained with a phosphotungstic acid solution 1 g/100 mL in sterile water, before the TEM analysis. The samples were dried for 1 min and then examined under TEM and photographed at an accelerating voltage of 64 kV.

### 2.5. Stability Study of NCs after Lyophilisation

The lyophilisation process of NCs provides an extended storage period at room temperature and can be carried out without the use of lyoprotectants because of the very low water content. The samples were frozen by a freezer (−23 °C) overnight before lyophilisation. Then, the samples were moved to a freeze-drier. The temperature was set to −23 °C and the pressure was −1.0 bar. The drying time was 24 h. The pressure and the temperature remained unchanged during the process.

The stability of the lyophilised NCs was evaluated after reconstitution of the colloidal system to the original volume with distilled water, using a vortex mixer at room temperature. The samples were stored in sealed glass containers after being placed into a desiccator containing silica gel to absorb water vapor. The samples were also protected from light. The stability of the lyophilised NCs was assessed by checking the size, ζ-potential, polydispersity and morphology every week for 2 months.

### 2.6. Stability Study of NCs in Gastrointestinal Media

NCs could be used to protect the entrapped compound from the effects of the gastrointestinal fluids. Accordingly, NC formulations were tested for their stability using simulated gastrointestinal conditions. Simulated gastric fluid (SGF) was used to investigate the gastric stability of NCs, as previously reported [23,24]. Briefly, 5 mL of NCs was suspended in 5 mL of SGF (0.32% *w*/*v* pepsin, 2 g of sodium chloride and 7 mL HCl dissolved in 1 L water and pH adjusted to 1.8 using 1 M HCl) and incubated at 37 °C under shaking at a speed of 100 strokes/min. After 2 h, the sample was collected. The size and PdI were analysed by DLS, while the morphology of the colloidal systems was analysed by TEM.

The stability of the samples was also investigated in simulated intestinal fluid (SIF) containing an intestinal enzyme complex (lipase 0.4 mg/mL, bile salts 0.7 mg/mL and pancreatin 0.5 mg/mL) and 750 mM calcium chloride solution at 37 °C, under shaking, with a speed of 100 strokes/min. The pH of the mixture was adjusted to a value of 7.0 with NaOH 0.1 N. After 2 h, the sample was collected and its physical and morphological properties were assessed by size and PDI analysis by DLS and TEM.

### 2.7. Preparation of Nanocarriers Based on AN and FITC

NCs were obtained from nanoliposomes (SUVs), which were prepared using the film hydration method. The nanoliposomes were formulated as follows: phospholipids (60 mg), cholesterol (20 mg) and AN (20 mg) or FITC (5 mg) were dissolved in dichloromethane/methanol mixture (20 mL of a mixture 3:2 *v*/*v*). The obtained organic solution was evaporated under vacuum to obtain a lipid film, which was hydrated by the addition of PBS (10 mL) using a mechanical stirrer (RW20 digital, IKA, Staufen im Breisgau, Germany) for 30 min in a water bath at a constant temperature of 37 °C for PC and 60 °C for PS. The resulting formulations were reduced in size using an ultrasonication probe for 3 min (two cycles of 90 s). During the sonication, the samples were kept in an ice bath to prevent lipid degradation. After that, a gentle centrifugation (1205× *g*, 1 min) was performed to remove possible metallic particles released during the ultrasonication. The NCs were prepared by the trapping method, according to Asprea et al. [20]. A 0.1 M solution of CaCl_2_ was added drop-by-drop to the liposomal suspension under magnetic stirring (150 rpm, at room temperature) until the formulation became cloudy, indicating the formation of NCs. The molar ratio between PC and CaCl_2_ was 1:1, while the molar ratio between PS and CaCl_2_ was 1:4.

### 2.8. Determination of Encapsulation Efficiency of AN–NCs by HPLC

After preparation of the NCs, free AN was removed by dialysis using bags with a pore size of 3.5–5 kD, and according to previous studies [25]. The dialysis bag was placed in 1 L of distilled water at room temperature for 1 h under stirring. The physical mixture was used as a control to validate the procedure. The AN-loaded content was quantified by HPLC–DAD analysis using a standard sample of AN, after the treatment of NCs with methanol to destroy the cochleates. HPLC–DAD analyses were performed with a HP 1200 Liquid Chromatograph (Agilent Technologies, Palo Alto, CA, USA), equipped with a Diode Array Detector (DAD), managed by a HP 9000 workstation (Agilent Technologies). The column was a Varian Polaris RP18 (250 mm × 4.6 mm i.d., particle size 5 μm) (Agilent Technologies) maintained at 27 °C. The chromatograms were acquired at 223 nm. The eluents were acetonitrile (A) and formic acid/water at pH 3.2 (B) at a flow rate of 1 mL/min. The following gradient profile was applied: 0–3 min, 10% A, and 90% B; 3–11 min, 10–38% A, and 90–62% B; 11–25 min, 38% A, and 62% B; 25–30 min, 38–50% A, and 62–50% B; and 30–34 min, 50–10% A, and 50–90% B. The post time was 10 min. The injected volume of the samples was 10–20 µL.

The calibration curve was obtained from a dilution series of the AN reference standard solubilised in MeOH, in the range between 56 and 0.56 ng/mL. Linear regression was used to establish the calibration curve. AN was quantified using the peak areas acquired at 223 nm. The correlation coefficient (*R*^2^) was 0.9995. The data are expressed as the mean ± SD of the three experiments.

The encapsulation efficiency (EE%) for each preparation was calculated using the following equation:

EE% = (*W*t/*W*i) × 100%, (3)

where *W*t is the total amount of the loaded AN and *W*i is the total quantity of AN added initially during the preparation. The encapsulation efficiency was determined in triplicate.

### 2.9. Determination of Encapsulation Efficiency of FITC–NCs by HPLC

Free FITC was removed by means of dialysis, as previously described. The contents of FITC were determined by the same HPLC instrument used for AN quantification. The column was a Lichrosorb RP18 (4.6 mm × 100 mm i.d., 5 µm) (Agilent Technologies) maintained at 27 °C. The mobile phases were (A) acetonitrile and (B) formic acid/water pH 3.2, at a flow rate of 0.8 mL/min and an injection volume of 10 μL. The following gradient profile was used: 0–5 min, 10–40% A, and 90–60% B; 5–10 min, 40–50% A, and 60–50% B; 10–12 min 50–55% A, and 50–45% B; 12–15 min, 55% A, and 45% B; 15–18 min, 55–90% A, and 45–10% B; and 18–20 min, 10% A, and 90% B. The post time was 5 min. The chromatograms were acquired at 224 nm. The linearity range of responses of FITC dissolved in CH_3_OH was determined on five concentration levels from 6.40 ng/mL to 520 ng/mL and the correlation coefficient (*R*^2^) was 0.9994 [26].

The encapsulation efficiency was calculated using the equation described in the previous paragraph. In this case, Wt is the total amount of the loaded FITC and Wi is the total quantity of FITC added initially during the preparation.

### 2.10. In Vitro Release Study

The in vitro release of AN from the NCs was investigated using the dialysis bag method. In order to simulate the physiological conditions, PBS (pH 7.4) and enzyme-free SGF and SIF were used as dissolution media. A total of 2 mL of AN–NCs suspension was deposited into the dialysis membrane (pore size 3.5 kD) and placed in 200 mL of the release medium. The temperature was set at 37 °C and the system was stirred at 150 rpm. Release into the PBS was monitored for 24 h while in SGF and for 2 h while in SIF, corresponding to the theoretical transit through the gastrointestinal tract; aliquots of one millilitre were withdrawn in duplicate and replaced with fresh dissolution medium. The samples were analysed by HPLC for the quantification of released AN. The percentage of AN released was calculated as follows:

(4) $$\%\mathrm{AN}\;\mathrm{released}=\left(\frac{{\mathrm{AN}}_{r}}{{\mathrm{AN}}_{\mathrm{tot}}}\right)\times 100,$$

where AN_r_ is the amount of AN detected by HPLC analyses and AN_tot_ is the total quantity of AN deposited into the dialysis membrane.

Furthermore, to evaluate the kinetics of drug release from the NCs, different mathematical models were used, i.e., zero order and first order kinetics model, the Higuchi model, the Korsmeyer–Peppas model and the Hixson–Crowell model. The best fitting model was selected according to the best regression coefficient (*R*^2^) value for the release data.

### 2.11. Cell Viability and Uptake Studies

The albino mouse embryonic 3T3 fibroblast cell line and the murine monocyte/macrophage cell line J774a.1 were used for cell viability and uptake studies [27,28]. The cell lines were maintained in Dulbecco’s Modified Eagle’s Medium (DMEM, Sigma-Aldrich) supplemented with foetal bovine serum, 100 units/mL penicillin, and 100 μg/mL streptomycin; for the 3T3 cell line, an additional glucose concentration (4.5 g/L) was used. The cells were maintained under standard culture conditions (37 °C, 5% CO_2_, 95% air and 100% relative humidity).

The cells were inoculated into 96-well microplates and maintained under standard culture conditions for 24 h to test the cell viability. Thereafter, the medium was replaced with fresh medium containing different concentrations of NCs or LPs. After 24 h, a WST-8 test was performed following the kit protocol as indicated by the manufacturer and as described in [28]. Briefly, 100 µL of DMEM supplemented with 10% WST-8 reagent was incubated in each well for 2 h at 37 °C. The formazan concentration was quantified by an optical absorbance at 450 nm, with a reference wavelength of 630 nm and by subtracting blank values. The data were expressed as a percentage of the optical absorbance with respect to the controls.

The cells were inoculated into a 33-mm petri dish and maintained under standard culture conditions for 24 h for uptake experiments. Subsequently, the medium was replaced with fresh medium containing different concentrations of FITC loaded in NCs or SUVs. After 1 h, the medium was removed and the cells were fixed in 3.6% PFA in PBS for 10 min at room temperature, stained with DAPI and analysed by confocal imaging. Images were acquired by a Leica SP7 confocal microscope and underwent no subsequent manipulation. A minimum of five different fields was acquired from each sample and all samples were performed in triplicate.

## 3. Results

### 3.1. Preparation and Characterisation of NCs

**Figure 2:**
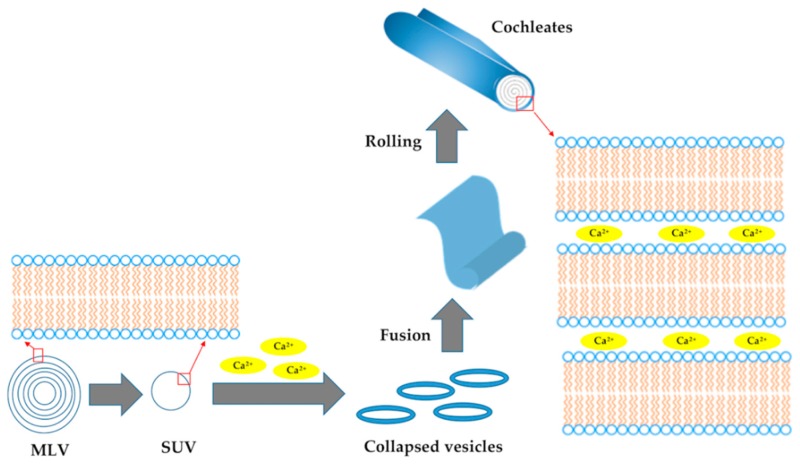
Multi-step preparation process of nanocochleates.

The NCs were prepared according to the multi-step preparation reported in Figure 2.

Briefly, as a first step, nanosized LPs were prepared according to the film hydration method using PC or PS and cholesterol, in the gravimetric ratio reported in the experimental part. The lipid film was dispersed in PBS to obtain MLVs. The formation of the SUVs was performed using an ultrasonication probe. In a further step, the SUVs collapsed after the addition of CaCl_2_ solution when added in the molar ratio 1:1 to PC liposomes (PC–SUVs) and in the molar ratio 4:1 to PS liposomes (PS–SUVs). Then, the collapsed vesicles fused giving large sheets, which rolled-up to give NCs. The calcium ions were essential for the stability of the system, and the aqueous phase in the structure of NCs was very limited, as reported in Figure 2. Both the SUV and NC formulations were characterised in terms of size, homogeneity and ζ-potential by dynamic and electrophoretic light scattering (Table 1).

Both PC–SUVs and PC–NCs had a narrow size of ca. 150 nm and they were highly homogeneous as evinced by the PdI (Table 1). Both the liposomes and the NCs based on PC were smaller than those prepared with PS. In particular, the PS–NCs were not homogeneous (Table 1). The dimension of the nanocarriers in the suspension was based on the measurement of their translational diffusion coefficient. This value is related to the length, L, of their major axis as described by Equation (1). The shape of the particles, but not the size, is linked by the geometrical coefficient, FD, which is 1 for spheres. However, it was determined for the NCs using a simplified geometry of long rods, according to Equation (2) [19,20]. All the nanovectors were negatively charged, and, as expected, the ζ-potential was a very low for the nanocarriers based on PS.

**Figure 3:**
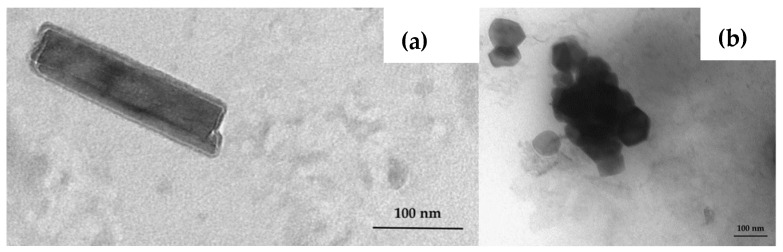
TEM images of PC–NCs (**a**) and PS–NCs (**b**) (scale 100 nm).

The morphological characterisation was completed by the observation of TEM pictures. The size and homogeneity of the liposomes based on PC and PS were confirmed (data not reported). The cigar-like shape of PC–NCs was strongly assessed (Figure 3a). PC–NCs dimensions were comparable with the dimensional distribution results obtained from the DLS analysis. The TEM images of PS–NCs confirmed the presence of polydisperse systems with structures different to NCs (Figure 3b).

### 3.2. Stability Study of Empty NCs

Firstly, the stability of the NCs was assessed by measuring the changes in terms of the average dimensions, polydispersity and ζ-potential values after the lyophilisation process and resuspension at room temperature with distilled water. The analysis was performed immediately after the lyophilisation process, which did not affect the physical characteristics, when re-suspended in water, as reported in Table 2. All the samples were reconstituted and analysed by DLS, ELS and TEM every week. It was only the PC–NCs that did not experience considerable modification in size, homogeneity and ζ-potential values (Table 2).

**Figure 4:**
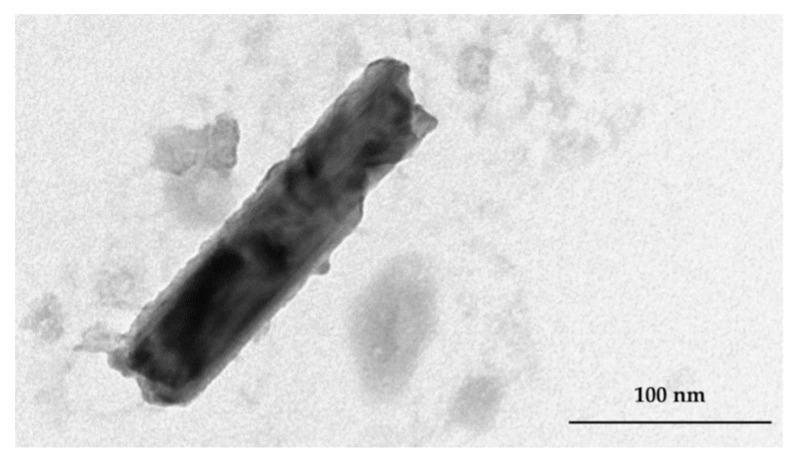
TEM image of PC–NCs re-suspended with distilled water after two months of storage at room temperature in the lyophilised state (scale 100 nm).

TEM analyses confirmed the dimensional data obtained by DLS concerning PC–NCs (Figure 4). Instantly after the preparation, PC–NCs had a dimension of 150 nm, while in the following 60 days their size increased by about 20 nm, while their ζ-potential values remained almost constant during this stability study. By contrast, the PS–NCs were not stable and their size increased by about 80 nm during storage. The TEM pictures showed the presence of aggregates (data not reported), confirming the results reported in Table 2.

### 3.3. AN–NCs and FITC–NCs Production

As a result of the stability testing of the two NC formulations, PC–NCs were selected as drug delivery systems to be investigated in the present study. AN or FITC was added to the lipid phase and their preparation was carried out using the same scheme reported in Figure 2.

FITC–LPs and FITC–NCs had a good size and homogeneity to test their performance for uptake in the macrophage J774a.1 cell line (Table 3). The average FITC-entrapment efficiency in the SUVs and NCs obtained by HPLC–DAD analyses was 87.5 ± 1.0 and 87.2 ± 0.1%, respectively.

**Figure 5:**
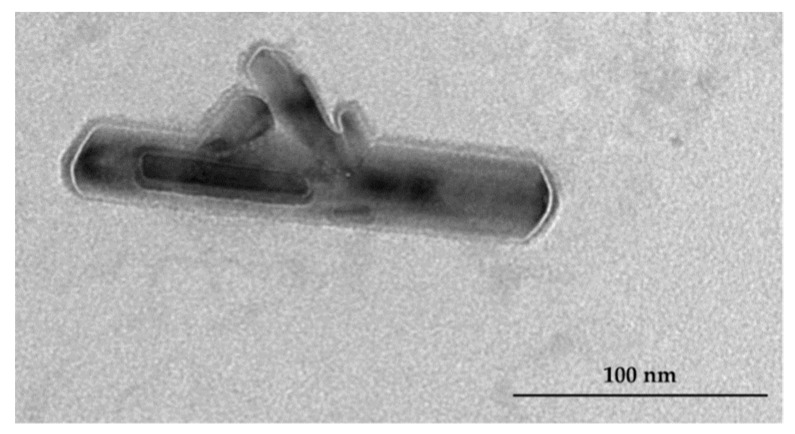
TEM image of AN–NCs (scale 100 nm).

The dimensions of the AN–NCs was ca. 150 nm, with a very low PdI, which resulted in suitability for all routes of administration, not only oral [29]. These data were also reflected by the TEM which exhibited NCs as tubular rod structures (Figure 5). The structure of the NCs is not modified in terms of size by AN loading, which means that AN does not interfere with the cohesion and packing of the apolar chains of the cochleate membrane. This is typical of small terpenes, which are able to decrease the size of lipid nanocarriers by forcing the PC structure to increase its surface curvature [30].

The average AN-entrapment efficiency in both SUVs and NCs was obtained by HPLC–DAD; the results were 71.1 ± 2.3 and 70.6 ± 5.9%, respectively (Table 3).

### 3.4. Stability of AN–NCs in Gastrointestinal Fluids

It is known that AN is not stable in the presence of gastrointestinal enzymes. Accordingly, one of the aims of this study was the development of a formulation able to protect the incorporated compound from degradation in gastrointestinal fluids. The gastrointestinal fluids may have an influence on the integrity of NCs. The physical stability of AN–NCs was assessed in SGF (pH 2) and in SIF (pH 7). These media did not affect their structure after two hours of incubation. The DLS analyses revealed that the mean diameter of the NCs was not affected by these conditions: after incubation in both gastro-enteric media, their mean size was 143 ± 1 nm with PdI 0.25 ± 0.02.

### 3.5. In Vitro Release Studies

After demonstrating the physical stability of NCs in gastrointestinal conditions, the in vitro release of AN from NCs was investigated by the dialysis bag diffusion technique. The test was carried out in both SGF (pH 2) and SIF (pH 7) for two hours and in physiological pH conditions (PBS, pH 7.4) for 24 h.

The percentage of AN released in SGF was only 2.31 ± 0.02%, while in SIF, it was 14.75 ± 1.14%. These results suggest that NCs may prevent AN burst release in the gastrointestinal tract, since about 85% of the compound remained entrapped in the NCs.

**Figure 6:**
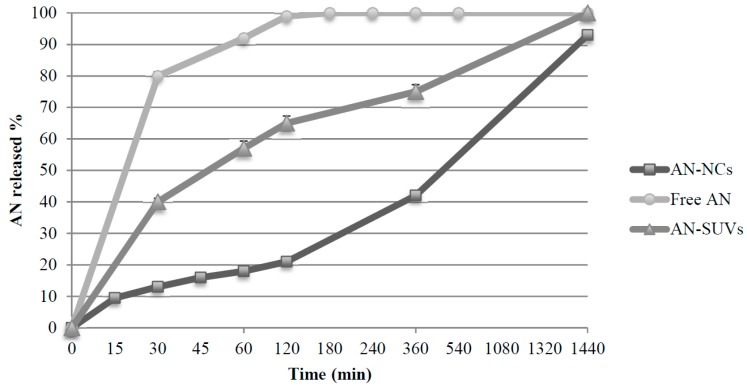
In vitro release profiles of free AN, AN–NCs and AN–SUVs in PBS. AN solution and AN–SUVs were tested to evidence the superiority of AN–NCs on the gradual release of AN. The data are displayed as the mean ± SD; *n* = 3.

In PBS, the release of AN from NCs was not immediate, but gradual, unlike in the case of free-AN, indicating that the formulation results in a more prolonged effect (Figure 6).

The AN release from NCs can be described as a biphasic process and the mathematical model of the drug release data was found to best fit the Hixson–Crowell release model: $W_{0}^{\frac{1}{3}}-W_{t}^{\frac{1}{3}}=K_{s}t$; where *W*_0_ is the initial amount of the drug in the pharmaceutical dosage form; *W_t_* is the remaining amount of the drug in the pharmaceutical dosage form, at time *t;* and *K_s_* is a constant, incorporating the surface–volume relation. The *R*^2^ was 0.9961. This model has been frequently used to describe drug release from several dosage forms with modified release. According to this model, the drug release is described by dissolution, characterised by the surface area and diameter of the particles. Consequently, based on the obtained results, it is possible to hypothesise that this behaviour may be due to the strong affinity of hydrophobic AN to the lipid structure of NCs**.**

### 3.6. Biocompatibility Studies

**Figure 7:**
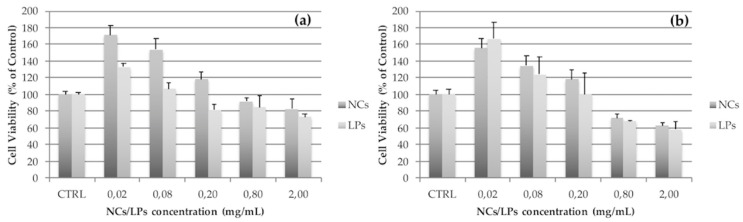
Cell viability after 24 h of exposition to NCs or LPs. The concentrations are expressed in mg/mL. The data represent the percentage of control ± SD. The J774a.1 (**a**) is a monocytes/macrophages cell line; the 3T3 (**b**) is a fibroblasts cell line.

The biocompatibility of NCs was tested using two cell lines: macrophage J774a.1 and fibroblasts 3T3. SUVs were used as comparable reference nanovesicles. As a colorimetric, non-radioactive assay, the WST-8 test was selected for assessing cell viability and proliferation because it indicates the mitochondrial activity and hence reflects the cell viability. WST-8, a highly water-soluble tetrazolium salt, is reduced to a soluble purple formazan derivative by trans-plasma membrane electron transport from NADH via an electron mediator. The concentration of formazan was quantified by optical absorbance at 450 nm, with a reference wavelength of 630 nm and by subtracting blank values. The mean value and the standard deviation are the results of nine measurements: the test was performed in three independent experiments and in each experiment, the samples were tested in triplicate. The data were expressed as the percent of optical absorbance with respect to the controls. As indicated in Figure 7, both SUVs and NCs showed no cytotoxicity at the concentration needed for massive uptake, namely, with a dilution of 1:40. Higher concentrations showed a decrease in cell viability, validating the dose–response curve. By contrast, it is remarkable that lower concentrations of the nanovesicles increased the cell metabolism rates, which was probably due to the active uptake process.

### 3.7. Cellular Uptake Studies

**Figure 8:**
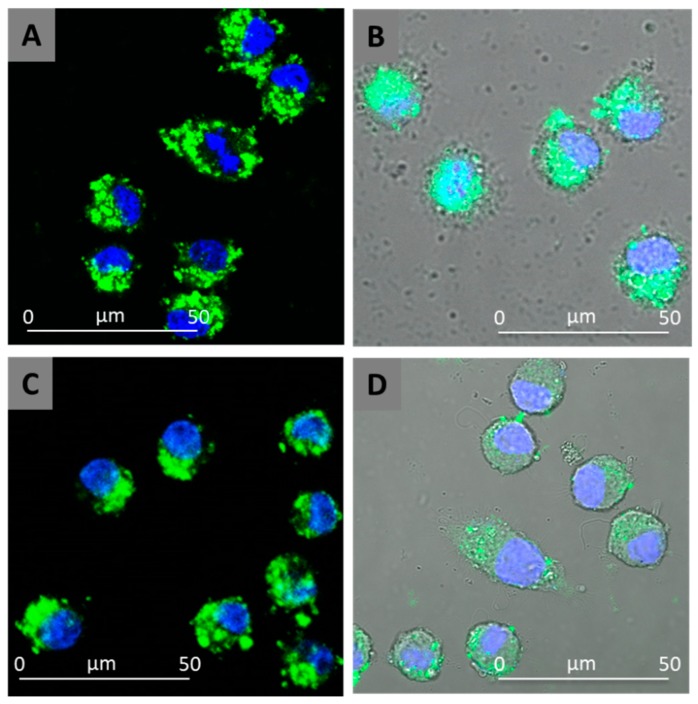
Confocal images of the macrophage uptake of NCs (**a**) and SUVs (**c**). Following nuclear staining with DAPI and FITC encapsulation into NCs and SUVs, the cell nuclei appear in blue and the NCs/SUVs appear in green. The confocal images are also superimposed to Bright Field acquisition (**b**,**d** for NCs and SUVs, respectively) to show the unaltered morphology of the cells and the localisation of intracellular nanocarriers.

In Figure 8, the uptake of both NCs and SUVs by macrophage J774a.1 cell line, using nanoparticles loaded with FITC (FITC–NCs and FITC–SUVs), is reported. The uptake was tracked by the green fluorescence of FITC using a confocal microscope (Figure 8).

As reported in Figure 8, massive uptake takes place but the fluorescence is typically in the cytoplasm without entering the cell nuclei. NCs and SUVs exhibit very similar uptake capability.

## 4. Discussion

In the present study, the potential of NCs is explored for the delivery of AN, a very promising active natural constituent with various potential therapeutic benefits, but due to the low bioavailability and instability in gastrointestinal media when administered with conventional dosage forms, it has never reached a milestone therapeutic potential. Accordingly, the development of suitable delivery systems for AN represents an urgent issue to formulate effective therapeutic approaches. Lipid-based delivery systems, especially vesicles, have attracted huge efforts as high bio-compatible and biodegradable nanocarriers crossing membrane delivery systems because of their resemblance to the cell membrane. One of the main drawbacks of conventional liposomes for oral administration is their poor stability in the gastrointestinal environment. By contrast, NCs can easily be lyophilised to obtain solid, stable, biocompatible and biodegradable nanovectors [1,2,5,6].

The NCs were simply developed from nanoliposomes (Figure 2), selecting both PS and PC and cholesterol as lipid phases due to their close resemblance to natural membranes and their high compatibility for human use. Ca^2+^ was selected among the diverse divalent cations to generate NCs because it can enhance membrane fusion and phagocytosis. It is well documented that calcium ions induce perturbations of the contact region and thereby promote the membrane fusion [11,31]. Astonishingly, in our studies, only PC and cholesterol generated monodisperse NCs with a tightly packed structure after the addition of Ca^2+^. As previously reported, PS-based NCs are not stable, producing systems with elevated polydispersity because of a tendency to form stable and huge aggregates, which represents a serious drawback at the industrial level [15]. By contrast, developed PC-based NCs were stable after lyophilisation and re-suspension in distilled water, and after incubation in simulated gastric and intestinal media. In vitro dissolution studies explained an extended release, making AN available over a prolonged period after administration. The PC-based NCs were biocompatible. Even at high concentrations, the cell morphology and vitality were not affected by internalisation. Moreover, high cellular uptake of PC-based NCs was found in macrophages using fluorescent nanovectors. After treatment of the cells with NCs, a bright fluorescent color of the cytoplasm arose due to the FITC and it was clearly distinguished from the nucleus stained with DAPI. Due to the similar uptake performances of SUVs and NCs, it is plausible that the developed NCs fuse with the cell membrane due to the interaction of calcium ions with the membrane containing negatively charged lipids, entering into the cells as nanovesicles [11]. A distinctive geometry, together with peculiar internal interactions, makes NCs ideal as pharmaceutical carriers, which may provide unparalleled protection for the molecular species in order to be carried harmlessly toward its destination. Developed NCs are inexpensive, stable, monodisperse, highly safe, biocompatible, and cell-permeating delivery systems. Moreover, they have high EE%, and suitable drug release properties for oral delivery, but with possible uses in other routes of administration. NCs are characterised by a series of solid-lipid bilayers; the components within the interior of this structure remain intact, even though the outer layers of NCs may be exposed to harsh external environmental conditions or enzymes. This interior structure of NCs is essentially free of water and resistant to penetration by oxygen, which leads to an increased shelf-life of the formulation. NCs can be stored at room temperature or 4 °C, and can be lyophilised to a powder form. Thus, NCs can be used to formulate capsules, pills, tablets, granules, suspensions or emulsions. Due to the ease of the internalisation process, this system could be exploited by employing future in vivo experiments and could be of interest in various therapeutic options.

## Figures and Tables

**Table 1:** Physical characterisation of empty liposomes and nanocochleates.

Sample	Size (nm)	PdI	*ζ*-Potential (mV)
PC–SUVs	150 ± 2	0.20 ± 0.02	−29.3 ± 0.9
PC–NCs	150 ± 2	0.24 ± 0.01	−21.6 ± 1.3
PS–SUVs	205 ± 37	0.25 ± 0.03	−37.2 ± 7.1
PS–NCs	207 ± 44	0.55 ± 0.05	−36.4 ± 1.4

PC–SUVs: liposomes made of phosphatidylcholine; PC–NCs: nanocochleates made of phosphatidylcholine; PS–SUVs: liposomes made of phosphatidylserine; PS–NCs nanocochleates made of phosphatidylserine. The data are displayed as the mean ± SD; *n* = 3.

**Table 2:** The particle size, polydispersity index (PdI) and ζ-potential of PC–NCs and PS–NCs as a lyophilised product after two-month storage at 25 °C.

**PC–NCs**	***t*_0_**	**After 30 Days**	**After 60 Days**
Size (nm)	150 ± 2	166 ± 5	172 ± 3
PdI	0.24 ± 0.01	0.25 ± 0.02	0.25 ± 0.01
ζ-Potential (mV)	−21.6 ± 1.3	−19.4 ± 1.1	−18.5 ± 1.0
**PS–NCs**	***t*_0_**	**After 30 days**	**After 60 days**
Size (nm)	207 ± 44	292 ± 22	280 ± 25
PdI	0.55 ± 0.05	0.53 ± 0.04	0.55 ± 0.05
ζ-Potential (mV)	−36.4 ± 1.4	−31.4 ± 2.1	−27.2 ± 1.1

PC–NCs: nanocochleates made of phosphatidylcholine; PS–NCs nanocochleates made of phosphatidylserine. The data are displayed as the mean ± SD; *n* = 3.

**Table 3:** Physical and chemical characterisation of AN- and FITC-loaded LPs and NCs.

Sample	Size (nm)	PdI	ζ-Potential (mV)	EE (%)
AN–SUVs	148 ± 2	0.13 ± 0.01	−27.5 ± 2.9	71.1 ± 2.3
AN–NCs	140 ± 1	0.22 ± 0.05	−22.3 ± 3.1	70.6 ± 5.9
FITC–SUVs	180 ± 2	0.20 ± 0.05	−29.2 ± 0.9	87.5 ± 1.0
FITC–NCs	177 ± 1	0.13 ± 0.02	−20.4 ± 2.3	87.2 ± 0.1

AN–SUVs: andrographolide-loaded liposomes; AN–NCs: andrographolide-loaded nanocochleates; FITC–SUVs: fluorescein isothiocyanate-loaded liposomes; FITC–NCs: fluorescein isothiocyanate-loaded nanocochleates. The data are displayed as the mean ± SD; *n* = 3.
